# Physical structure and biological composition of canopies in tropical secondary and old-growth forests

**DOI:** 10.1371/journal.pone.0256571

**Published:** 2021-08-20

**Authors:** David B. Clark, Steven F. Oberbauer, Deborah A. Clark, Michael G. Ryan, Ralph O. Dubayah

**Affiliations:** 1 University of Missouri-St. Louis, St. Louis, MO, United States of America; 2 Dept. of Biological Sciences, Florida International University, Miami, FL, United States of America; 3 Fairchild Tropical Botanic Garden, Miami, FL, United States of America; 4 USDA Forest Service, Rocky Mountain Research Station, Ft. Collins, CO, United States of America; 5 Affiliate Faculty, Department of Forest, Rangeland and Watershed Stewardship and Graduate Degree Program in Ecology, Colorado State University, Fort Collins, CO, United States of America; 6 Department of Geography, University of Maryland, College Park, MD, United States of America; Chinese Academy of Forestry, CHINA

## Abstract

The area of tropical secondary forests is increasing rapidly, but data on the physical and biological structure of the canopies of these forests are limited. To obtain such data and to measure the ontogeny of canopy structure during tropical rainforest succession, we studied patch-scale (5 m^2^) canopy structure in three areas of 18–36 year-old secondary forest in Costa Rica, and compared the results to data from old-growth forest at the same site. All stands were sampled with a stratified random design with complete harvest from ground level to the top of the canopy from a modular portable tower. All canopies were organized into distinct high- and low-leaf-density layers (strata), and multiple strata developed quickly with increasing patch height. The relation of total Leaf Area Index (LAI, leaf area per area of ground) to patch canopy height, the existence of distinct high and low leaf- density layers (strata and free air spaces), the depth and LAI of the canopy strata and free air spaces, and the relation of the number of strata to patch canopy height were remarkably constant across the entire successional gradient. Trees were the most important contributor to LAI at all stages, while contribution of palm LAI increased through succession. We hypothesize that canopy physical structure at the patch scale is driven by light competition and discuss how this hypothesis could be tested. That canopy physical structure was relatively independent of the identity of the species present suggests that canopy physical structure may be conserved even as canopy floristics shift due to changing climate.

## Introduction

The capacity of any forest to take up CO_2_ is directly related to the total leaf area and its distribution in three dimensions [1–4], and to the photosynthetic capacities of the species contributing this leaf area [5]. Changes in any of these factors on a given landscape could potentially have major impacts on net CO_2_ flux. Tropical rainforests are particularly significant in this regard, because they are major reservoirs of the planet’s terrestrial carbon [6], process a significant portion of global net primary productivity [7–9], and contain the heaviest concentration of terrestrial biodiversity [10]. Old-growth tropical rainforests [11] are rapidly being converted to alternative land uses [12, 13], and the consequences of these conversions for global cycles of carbon and water are poorly understood [14].

One of the principal results of old-growth removal in the tropics is the increasing area of secondary forests [12, 15–17], that is, forests regenerating after conversion of forests to agriculture or pasture [18]. Some biological characteristics of tropical secondary forests are well known. Changes in stem density and mortality rates as well as species replacements along chronosequences from regenerating pasture to old growth have been relatively well studied [15, 19–25]. Total plant biodiversity is generally lower and physical structure simpler than in old-growth forests, and these differences diminish with increasing time since stand initiation [11, 22, 24, 26].

In contrast, there is much less of quantitative information on the structure of forest canopies along the same gradient [15, 27]. This is true both for canopy physical structure, that is the 3-dimensional organization of leaves, branches and stems, and for canopy biological structure, ie. the species or functional group identity of these elements [28, 29]. While studies on canopy physical structure using remotely-sensed data are increasingly common [30–33], to date these have not to our knowledge been validated by direct harvest in older, taller secondary forests.

Budowksi [20] stated that young secondary forests are short with one canopy layer, and with time add more layers and increase in height until reaching three “increasingly difficult to discern” strata in 20–50 year-old secondary forests. In his influential text Richards [34] popularized the concept that old-growth tropical rainforest canopies are generally stratified into alternating layers of clumped leaves and relatively leafless space. In the first landscape-scale test of Richard’s ideas [35], we carried out ground to top-height harvests of all foliage at many points over an old-growth landscape in Costa Rica. We found that at small spatial scales (patches ~ 5 m^2^) canopies were ubiquitously stratified into alternating layers of high and low leaf density, and that the total number of strata was highly predictable from forest height at that spot.

In the research reported here we expanded the landscape-scale sampling with our complete harvest approach to cover the successional gradient from young secondary forests regenerating after pasture to old-growth tropical rainforest. Our primary goal was to use harvest data from secondary forest stands to assess the development of patch-scale physical and biological canopy structure across this successional gradient. We also wanted to determine if the highly predictable patterns of total leaf area and canopy stratification that we found in old growth applied to the younger, smaller, and less biodiverse secondary forests at this site.

We asked three central questions:

1. What physical and biological factors determine the total Leaf Area Index (LAI, m^2^ of total leaf area per m^2^ of ground) through tropical rainforest succession following pasture abandonment? Do tropical secondary forests support more or less LAI at a given height the old-growth forests?
2. Are tropical secondary forest canopies organized into clear strata, as proposed by Budowski [20] and Richards [34], or are these simpler forests organized differently?
3. Does the representation of different plant functional groups in forest canopies change through succession? We assumed that it would but wanted to analyze this from the point of view of total leaf area instead of the commonly-used metrics such as stem number, basal area or species diversity.

As the work progressed it became clear that the physical structure of the secondary forest canopies we sampled was remarkably similar to nearby old growth. These findings raised fundamental questions about the processes that generate and maintain canopy structure at patch scales. We discuss possible routes to the development of canopy organization from tropical secondary forests to old growth, and suggest how these hypotheses could be tested with a variety of data in future research. Lastly we consider how the patterns of canopy structure reported here might be affected by plant species changes resulting from global climate change.

## Materials and methods

The study area was the La Selva Biological Station in the Atlantic lowlands of Costa Rica. The old growth is Tropical Wet Forest in the Holdridge system [36]. Mean annual temperature is 25^o^ with annual rainfall averaging 4354 mm [37]. Detailed information about La Selva soils, plant and animal communities can be found in [38].

Between March and June 2005 three stands of differing ages were sampled (S1 Fig). The three sites regenerated from pasture abandonment approximately 18, 25 and 36 years prior to the study. We chose these specific sites to sample relatively young secondary forests with well-documented site histories (see below). The total number of sites was determined by the funds available for this specific research. The approximate ages and land uses of the sites were established using the remote sensing archives of La Selva (aerial photos from 1966, ‘71, ‘76, ‘83, and ‘88, and IKONOS satellite data from 2000). Documentation of the site histories is given in S1 File. In all stands the total leaf area was harvested from forest floor to canopy top using a modular 2.45 × 1.86 m^2^ footprint canopy tower [35]; tower sections were 1.86 m tall. Tower construction and vegetation sampling required an average of 4 days per tower with a field crew of five construction worker and one field technician.

We term the spatial scale of one tower footprint as a forest “patch” to emphasize the small spatial scale (ca. 5 m^2^). We refer to the height of the highest leaves harvested in a patch as “patch canopy height”.

Within each of the secondary forest stands, locations for potential tower constructions sites were selected using a random-number table to generate X and Y coordinates. Prior to visiting these sites we developed a stepwise protocol for site evaluation. Criteria for rejecting a site were: the presence of pre-disturbance remnant trees in the tower footprint; site in a stream or on a slope >11*; site within 40 m of another tower site; site in a recent tree-fall gap with maximum vegetation height < 5 m. The distance from a tower to its closest neighbor averaged 106 m (S1 Table). Because we intentionally avoided canopy gaps [39], the secondary patches sampled here should be considered samples of the main canopy heights in each stand, not a strictly random sampling of all possible canopy heights as in [35].

Harvested leaves were separated into four plant functional groups: trees; palms; lianas (woody vines); others (herbs, epiphytes, ferns, non-woody vines). The leaf area per functional group per tower section was measured in the laboratory using a LI-COR-3100 leaf area meter. From a carbon uptake point of view, the top LAI unit (highest in the canopy) is the most highly illuminated and the most productive unit of LAI [40]. For this reason we also analyzed the functional group composition of only the top LAI.

We compared the data from the secondary forest stands with data from a previous study of old-growth forest canopy structure using the same methods as [35]. In that study 45 patches in old growth were selected by a stratified random design based on soil phosphorus (0–10 cm depth) and degree of slope. For analyses involving forest height we included an additional 10 old-growth patches that were selected with a random protocol to have canopy heights <16 m; we did not include these sites in floristic analyses since they were not randomly sampled across the old-growth landscape.

Canopy strata were defined as in [35], ie successive vertical segments comprised of multiple adjacent tower sections, each section with an LAI >0.200, or if the stratum was a single tower section, with LAI >0.300. These criteria divided the data into two classes that differed in mean LAI by more than an order of magnitude. This definition of canopy strata extends the Connell et al. presence-absence model [41] with a continuous response variable. It is similar to using a threshold in LiDAR data to define open and closed sub-canopy space [42]. This definition of canopy strata also corresponds to Definition 7 of Parker & Brown [43], “Stratification = Clumped Leaf Area with Height.” We call the very low leaf density spaces between strata “free air space”.

All statistical analyses were carried out in JMP© version 14.3.0 (SAS Institute Inc. 2018). For ANCOVA analyses we followed the sequence of steps recommended in [44], including checks for linearity, outliers, equal variance and normally-distributed residuals.

Raw leaf-area data for the secondary forest stands are given in S2 Table, and raw data for the old-growth towers are published at https://doi.org/10.1111/j.1461-0248.2007.01134.x.

## Results

### LAI, canopy height and canopy stratification

Leaf area and canopy height were significantly lower in the 18-year-old secondary forest than in the 25 and 36-yr old stand (Table 1). The two older secondary forest stands were similar in height to the old-growth random sites, perhaps partly due to the different sampling protocols; in the secondary forests we avoided canopy gaps, while in old growth sampling was strictly random with respect to canopy height and thus included recent and regenerating gap sites.

**Table 1:** Leaf area index and canopy heights based on leaf harvest sampling across a successional gradient at the La Selva Biological Station, Costa Rica.

		Leaf Area Index (m^2^)				Canopy Height (m)			
Secondary Forests (SF)									
Site	N	Mean	Min	Max	SEM	Mean	Min	Max	SEM
**18-yr SF**	5	3.97^a^	2.99	6.01	0.55	10.8^a^	3.7	16.7	2.2
**25-yr SF**	4	7.21^b^	5.58	8.37	0.62	24.2^b^	22.3	26.0	0.8
**36-yr SF**	7	6.45^b^	4.14	8.60	0.69	27.9^b^	20.5	37.2	1.9
**Old growth (OG)**									
**OG low canopy**	10	2.99	0.33	6.86	0.63	8.9	1.9	16.7	1.4
**OG Random points**	45	6.00	1.20	12.94	0.32	27.2	3.7	44.6	1.1

SEM = Standard Error of the Mean. Means identified by the same superscript letter were not significantly different (ANOVA P>0.05).

In general LAI increased with increasing canopy height in all secondary stands. As Clark et al. found [35] for old growth at La Selva, the overall increase in LAI with height in secondary forests was actually composed of two separate patterns. LAI was added with increasing height in a predictable pattern up to approximately 23 m (r^2^ = 0.56, N = 7), but above this height there was no predictable relation between LAI and patch height (r^2^ = 0.08, N = 9).

**Fig 1:**
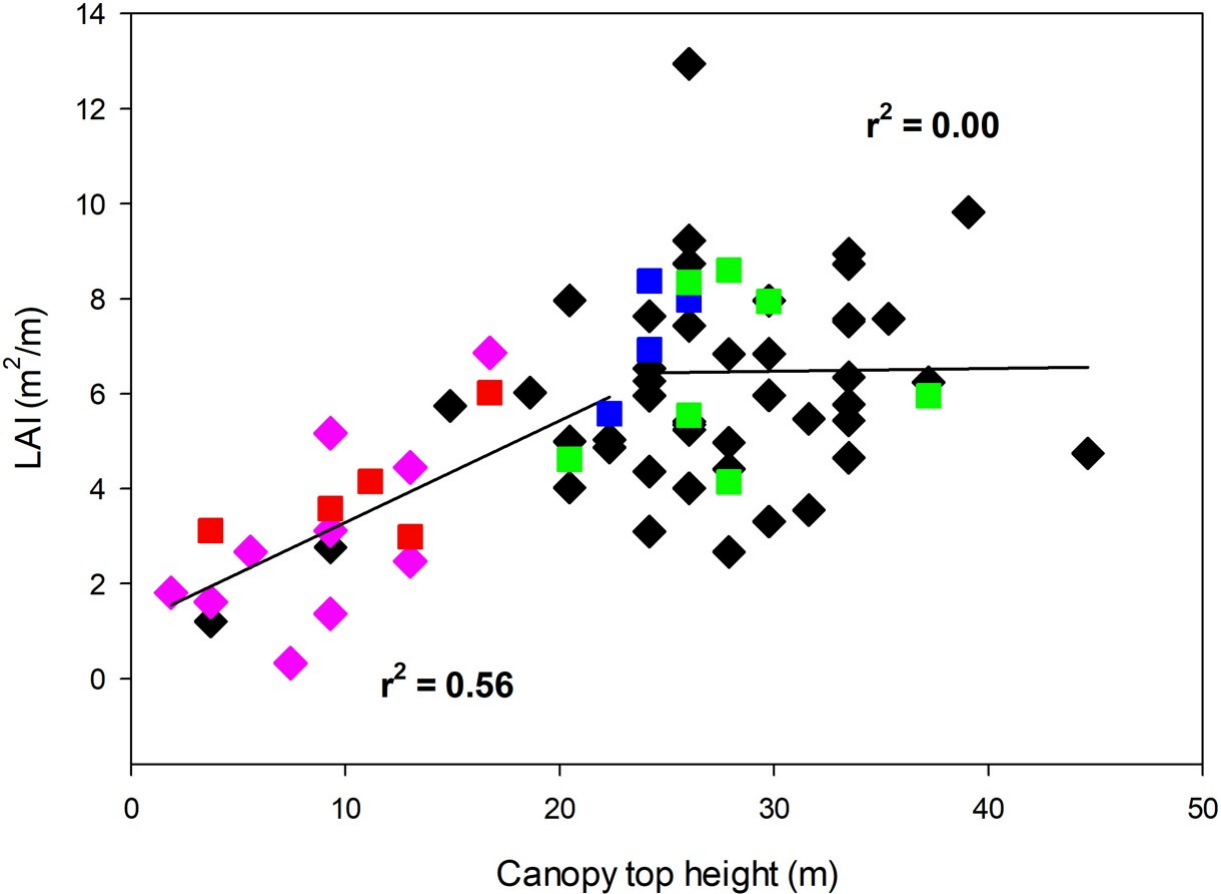
LAI as a function of canopy height across a successional gradient from abandoned pasture to old growth tropical wet forest at La Selva Biological Station, Costa Rica. Data from 18-yr-old secondary forest are shown in red squares, 25-yr-old sites in blue squares, 35 yr-old sites in green squares, black diamonds are 45 random patches in old growth, and pink diamonds are 10 old-growth patches selected for canopy height <16 m. R^2^ were calculated using towers from all sites that met the height criteria (N = 26 <23 m patch height, 45 > 23 m patch height). Canopy height ranges for the regressions were determined by visual inspection, so no probability values are given.

To assess the generality of LAI-height relations across the entire successional gradient, we combined all secondary forest stands and the two old-growth data sets (from randomly-located patches and from patches selected for low canopy, [35]). Canopy height appears to be the major driver of leaf area accumulation at this spatial scale in all stands up to ca. 23 m canopy height and an LAI of approximately 6 (Fig 1). Regardless of stand successional status, leaf area was added in a predictable linear fashion (r^2^ = 0.56) with increasing canopy height to ca. 23 m, above which there was no relation (r^2^ = 0.00) at this spatial scale (5 m^2^).

**Fig 2:**
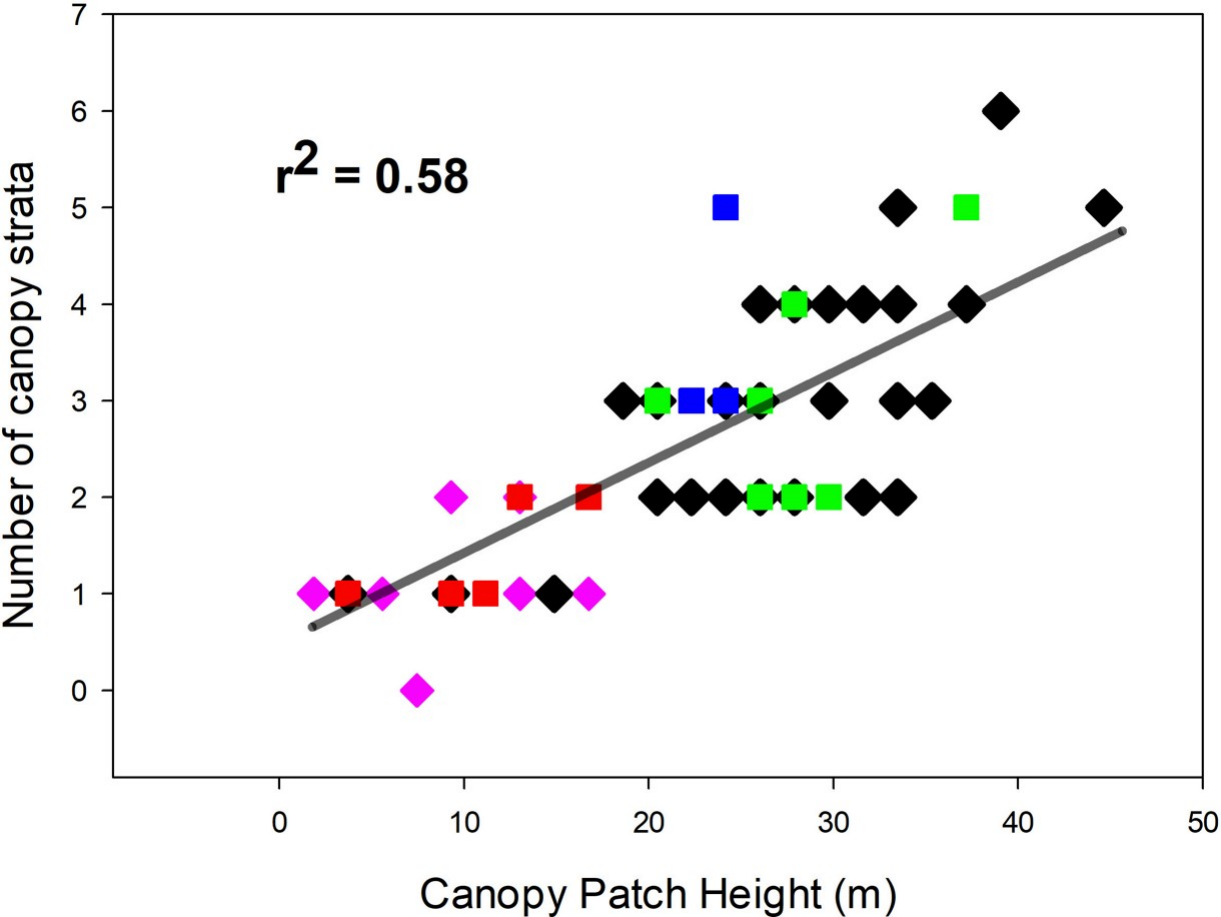
Number of canopy strata as a function of canopy height across a gradient of tropical forest succession at La Selva Biological Station, Costa Rica. Data from 18-yr-old secondary forest are shown in red squares, 25-yr-old sites in blue squares, 35-yr-old sites in green squares, black diamonds are 45 random patches in old growth, and pink diamonds are 10 old-growth patches selected for canopy height <16 m.

There were no significant differences among secondary forest stands in the depth or total LAI of strata and free air spaces (all four ANOVA P>0.05, Table 2), and there were no differences in these variables between all secondary forest sites combined and all old-growth sites (all four ANOVA P>0.05). In contrast to the saturating relation of LAI accumulation with forest height, the number of secondary-forest canopy strata increased as canopy height increased (r^2^ = 0.48, P_1-tail_<0.002, N = 16, S2 Fig). The relation of the number of strata to canopy height was not significantly different between all 55 old-growth sites and all 16 secondary sites (ANCOVA P>0.33). For all secondary and old-growth sites combined (Fig 2), the number of strata increased linearly with patch height (r^2^ = 0.58, P<0.001, N = 71). No site less than 10 m tall had more than one strata, and no site taller than 20 m had fewer than 2 strata (Fig 2).

**Table 2:** Canopy strata and free air space characteristics in secondary habitats and old growth at the La Selva Biological Station, Costa Rica.

	Strata				Free Air Spaces			
Secondary Forests (SF)	N	Mean LAI	SEM	Median Depth	N	Mean LAI	SEM	Median Depth
**18-yr SF**	7	2.72	0.60	3	6	0.14	0.04	1.5
**25-yr SF**	13	2.14	0.45	2	9	0.11	0.03	2
**36-yr SF**	21	2.05	0.33	2	16	0.13	0.03	2.5
**All SF**	41	2.19	0.24	2	31	0.12	0.02	2
**Old growth**								
**All random and LCH**	141	2.00	0.136	2	87	0.18	0.02	2

Definitions of strata and free spaces are given in **Methods**. Strata and Free Air Space depths were quantified as the number of canopy tower sections (each 1.86 m tall) occupied. Old-growth data [35] include all 45 randomly-selected sites and 10 sites selected for canopy heights <16 m (Low Canopy Heights LCH). SEM = Standard Error of the Mean.

The median number of plant functional groups per strata was 2, and >85% of strata contained > 2 functional groups across all secondary sites. Given our broad definition of plant functional groups (trees, palms, lianas, others), it is clear that strata were typically composed of more than one species and thus were not a simple consequence of one plant’s branching.

### Changes in plant functional group composition with tropical forest succession

Although patch-scale physical structure of the canopy was strikingly similar across this successional gradient (Figs 1 & 2, Table 2), there were substantial differences in the functional group composition of LAI along the same gradient (Table 3). There were no palms in the 18-year old secondary forest, and the contribution of palms to total LAI increased across the chronosequence. Even in the 36-yr-old secondary forest, however, palms were only 11% of total LAI, compared to 25% in old growth (Table 3). The “Others” category of functional groups (primarily herbs) decreased across the chronosequence. The combination of increasing palm LAI and decreasing contributions from other functional groups meant that the percentage of tree LAI changed relatively little across the successional gradient. Trees were always the most important functional group, making up 54–66% of total LAI in all stands.

**Table 3:** A. The relative contributions of major plant functional groups to leaf area (% + 1 SEM) across a tropical rainforest successional gradient at the La Selva Biological Station, Costa Rica. The “Others” category includes herbs, epiphytes, ferns and non-woody vines. Data from old growth are from [35]. B. Mean percentage functional group composition of the first (topmost) LAI unit (+ 1 Standard Error of the Mean).

	A. Mean percentage of total leaf area					B. Mean percentage of topmost LAI unit			
Site	N	Trees	Palms	Lianas	Others	Trees	Palms	Lianas	Others
18-yr old secondary forest	5	58.4 + 15.0	0.0 + 0.0	15.7 + 8.8	25.9 + 16.9	66.2+17.5	0.0+0.0	13.5+8.9	20.3+18.9
25-yr old secondary forest	4	56.3 + 14.5	4.6 + 3.0	24.5 + 14.2	14.6 + 4.5	93.6+3.9	0.0+0.0	6.1+4.0	0.3+0.2
36-yr old secondary forest	7	66.1 + 11.9	10.8 + 5.6	9.2 + 3.7	13.9 + 2.9	83.9+5.9	0.0+0.0	15.8+6.0	0.3+0.2
Old-growth random points	45	53.7 + 2.7	25.0 + 2.5	9.7 + 1.8	11.6 + 1.2	69.5+4.6	7.2+2.9	19.7+4.0	3.6+1.2

In terms of plant functional group composition, the top LAI showed a different pattern than that of total LAI across the chronosequence (Table 3). While trees were the dominant element of the top LAI in all stands, the percentage contribution of trees to top LAI was considerably higher in the middle-age successional forests than in 18-yr old forest or old growth. The top LAI of the 18-yr old forest was 20% non-woody vegetation, but this group was virtually absent in the older secondary forests and <4% of old-growth top LAI. Palms did not occur in the top LAI samples in any secondary stand.

## Discussion

### Regularity of canopy physical structure across succession–why?

The most surprising finding from this study was the consistency in canopy physical structure across a successional gradient ranging from 18-, 25- and 36-yr old secondary forests regenerating from pastures to old growth. The similarities in physical structure between young forests and old growth included omnipresent alternating strata of high- and very low-leaf-density strata, the predictable relation of total LAI and the number of the strata to patch canopy height, and the depth and leaf density of the strata and free air spaces. These similarities in canopy physical structure are counterintuitive given the different possibilities for increases in canopy patch height in secondary forest and in old growth. At the stand scale, young tropical secondary forests are characterized by spatially-extensive very rapid height growth (Table 1), whereas in old growth mean stand height is essentially constant and rapid height growth is primarily restricted to tree-fall gaps [45, Figs 7 and 9].

What processes could lead to the development of similar small-scale patch structure in stands of such different ages and developmental possibilities? We hypothesize that patch physical structure across all stand ages is fundamentally driven by competition for light, as suggested in a somewhat different context by Terborgh [46]. Neighboring individual plants grow upward or laterally into a stratum until light levels become too low to maintain a positive carbon balance for leaves at the bottom of the stratum. Over time, leaves are added at the top of the stratum and leaves at the bottom are shaded out and dropped, resulting in a net upward movement of the stratum. Ground-level strata are a special case, since the capacity for upward growth of many shade-tolerant life forms and species is limited. As the ground level stratum increases in depth, small-statured shade tolerant individuals may survive by dropping leaf area and waiting for higher light conditions [47, 48]. Intercalation of additional strata between the top stratum and the ground stratum we believe to be dependent on the availability of lateral diffuse, reflected and direct light [35]. This light will enter laterally through nearby canopy gaps as well as from spaces between neighboring leaves and strata.

The profile data from these secondary stands as well as from the prior direct harvest study of the adjacent old growth [35] provide support for these ideas. In all stands the mean and minimum canopy patch height for a given number of strata increased with canopy height (Fig 2), implying a general physical limit to the number of strata possible in a patch of a given canopy height. The fact that >85% of all strata contain at least two plant species (see Results) establishes that the conditions for intra-stratum light competition among individuals are widely present. Light levels drop rapidly with depth within a stratum [35], consistent with the possibility of creating photosynthetically-unprofitable zones at the bottom of strata after a certain strata thickness is attained. Also 1/3 of the vertical gradients in light between strata in old growth at La Selva were “reversed” gradients, with light increasing towards the ground from the stratum immediately above [35]; such reversed gradients can only be due to light entering from neighboring openings in the canopy. The data of Fauset et al. [49] (their Fig 2) also show reversed gradients among all the habitats they studied (intact forest, logged forest, secondary forest, forest fragments in SE Brazil). In contrast, in the 9- and 32-year-old forests studied by Matsuo et al. [50] their Fig 1 shows no reversed gradients amount 32 vertical light profiles in Mexican secondary forests (16 profiles per stand). Different methods were used in the Costa Rican, Brazilian and Mexican studies, so the reason for the differences among studies is unresolved at this point.

A notable finding from this study is the very rapid appearance of strata in the secondary forest habitats. Canopy patches with up to five strata were present in both the 25- and 36-year-old forests. This shows that strata can be organized relatively quickly in the life of a stand, but does not give any information about stratum longevity. It is useful to consider what plant types and plant subunits make up a canopy stratum at different heights at this small spatial scale. In the understory a stratum can contain whole plants as well as small tree seedlings and saplings. The topmost stratum is composed mainly of small branches of the tallest trees at that point, as well as portions of individual liana canopies (Table 3). Intermediate strata consist of branches of subcanopy trees, major portions of the crowns of smaller trees, and/or sections of large palm leaves. We hypothesize that the rapid appearance of more than two strata with stand age is due to the dynamic and relatively short-lived nature of the elements that compose intermediate-level strata. Our harvest data suggest that the necessary condition for at least one intermediate stratum to be formed is a patch canopy height of ca. 19 m (Fig 2), a criterion exceeded by all 25- and 36-yr old patches we sampled (Fig 1).

The minimum height requirement for a third and subsequent strata presumably reflects the vertical distance between the canopy and ground strata that is necessary for lateral light in the free air space beneath the upper-most stratum to reach a level that is photosynthetically profitable for plants to branch leaves laterally into the subcanopy space, or for plants beneath the nascent stratum to grow upward from below.

The lack of a relation between total leaf area and patch canopy height for taller patches may be related to the dynamic nature of the strata intermediate between the ground and top-most strata. Across the successional gradient total LAI was closely related to patch canopy height when there were only 0–3 strata (Figs 1 and 2). However as the potential for adding additional strata increases, the relation of patch LAI to patch height disappears. We hypothesize that this is due to the lags in strata reorganization as the top-most stratum increases in height or as lateral light environments change.

Both static and longitudinal data are needed to test these hypotheses. In our studies we sampled a range of stand ages and soil types over one tropical forest landscape. Static data (samples at one time) from additional tropical rainforest successional gradients are needed to determine the generality of these results.

Longitudinal data (following individual patches through time) are necessary to assess our hypotheses about stratum creation and maintenance. Two key environments to sample are very young secondary forests and regenerating gaps in old growth. Given the speed of upward and lateral growth in tropical forests (Table 1; [45, 51, 52]), one-stratum patches will grow to two-strata conditions in only a few years. If our hypotheses are correct, light levels at the bottom of the top stratum will decrease over time, becoming too low to support palm fronds or the leaves of most tree saplings. In contrast, as the top stratum grows upward, we predict that light levels in the subcanopy free air space will increase over time, and an intermediate stratum will then be formed by lateral ingrowth or upward tree growth. This point on the La Selva landscape is when canopy patch height exceeds ca. 19 m (Fig 2), but that limit could be different in other climate and successional conditions, or with different regimes of disturbance and/or forest height.

Testing these hypotheses will require repeated concurrent sampling of LAI and light levels in vertical canopy transects. There are two general approaches to sampling LAI [53]. Direct sampling involves physically harvesting leaves. Direct sampling gives detailed information on the 3-dimensional distribution of foliage and branches, and all LAI is completely sampled. Direct sampling is however expensive, and for obvious reasons is sampling is not suitable for long-term study of vertical transects.

An alternative is LAI estimation using remote sensing instruments [31]. These methods also offer the potential to map entire landscapes and regions, and will be the only way to obtain global coverage of tropical forest canopy structure. Remote sensing technologies that provide measures related to canopy structure include active sensors such as radar and lidar [33, 54–56], as well as sensors that rely on reflected and transmitted solar radiation such as the MODIS instrument and the Li-Cor LAI-2000. The physical principles behind all of these sensors are well known, and their capacity to indirectly estimate LAI in a variety of environments has been validated. Landscape-scale calibration of the methods in tropical rainforests, that is, an assessment of the absolute accuracy using directly-sampled ground data, is however lacking, and rarely been attempted (with MODIS [35]; hemispherical photography and LiCorr Plant Canopy Analyzer [30]; waveform lidar [57]). The most promising avenue for advancing measurement and understanding of detailed canopy structure and dynamics will be research combining indirect measurements with direct measurements for calibration, for example ground-based direct sampling coupled with ground-based LiCor LAI 2000 or terrestrial lidar and air-borne lidar sampling at a spatial resolution that can be matched to the ground sampling. An additional advantage of this data fusion approach is that it offers an avenue towards measuring spatial autocorrelations in canopy structure at scales larger than a tower footprint. An example of landscape-scale application of this approach is Tang et al. [57]. They calibrated waveform lidar over 55 LAI harvest sites at La Selva [35] and found an r^2^ of 0.42 between harvest and modelled LAI. These results suggest that is feasible to coordinate ground and remotely-sensed sampling to develop highly-predictive models to scale up results from spatially-limited ground sampling of LAI.

Static and long-term measurements of light levels within tropical rainforest canopies is even more difficult than accurately assessing LAI. Similar to LAI, light levels can either be measured directly or estimated indirectly. Unlike LAI, light levels vary both regularly within a day and irregularly due to clouds. Single-measurement or long-term vertical light transects can be done raising a light meter on a tall pole [49, 50], off canopy towers, at canopy crane sites or using subcanopy drones. There are practical issues with all of these methods for sampling light within canopy strata. As with LAI, research combing extensive indirect estimates with more limited direct measurements for calibration offers a path to more extensive spatial coverage.

### Biological structure vs. physical structure of canopies

The change in plant functional group composition of total LAI through secondary succession was largely expected based on previous research [24, 26, 58]. Trees contributed the greatest percentage of total and top LAI in all stands. Tall grasses and other non-woody species were only important in the 18-yr old stand, and palms became a larger percentage of total LAI through succession [19, 25]. This substantial change in the identity and diversity of species and functional groups across this successional gradient makes the consistency in canopy patch physical structure all the more remarkable. Species identity or diversity did not appear to significantly influence the processes that led to the physical organization of canopy patches organization at this site.

### The ecological significance of patch-scale canopy structure and dynamics

This research has led to a series of predictions about factors controlling the magnitude and vertical distribution of patch-scale leaf area in tropical rainforests across a successional gradient from young secondary forest to old growth. Our results come from one mesoscale TRF landscape, and research at other sites is necessary to evaluate the generality of our findings. If the regular patterns of canopy organization observed at this site are found to apply generally to tropical rainforests, this suggests that forest physical structure may be more buffered to future climate changes than are individual plant species. There is ample reason to be concerned about species, community and biome shifts that are currently under way in tropical rainforest due in part to changing global climate [59–62]. Within the tropical rainforest biome however, if physical structure is driven by the relatively species-independent processes that we hypothesize here, patch-scale canopy physical structure could change relatively less than biological structure.

Patch-scale canopy structure, the 3-dimensional physical and biological structure of forests at a spatial scale less than or equal to the crown area of an average canopy tree, is a fundamental organizational scale for any forest landscape. Patch structure reflects biophysical and biodiversity gradients, site history, disturbance regimes, and successional trajectories. Our research has shown that the direct measurement of patch-scale canopy structure can lead to new, testable hypotheses about the origin and maintenance of canopy structure across a large range of successional stages in tropical forests. Extending this research approach to other tropical-forest regions could greatly deepen our understanding of fundamental processes of forest ecology in tropical landscapes.

## Supporting information

S1 Fig Map of study area. (DOCX)

S2 Fig Secondary forest canopy strata. (DOCX)

S1 File Land use history of harvest sites as determined from remotely-sensed data. (DOC)

S1 Table Secondary forest tower UTM locations. (CSV)

S2 Table Raw data for all LAI harvest sites. (CSV)
