# Supplementary material for: Physical structure and biological composition of canopies in tropical secondary and old-growth forests
Source: PLoS One. 2021 Aug 20;16(8):e0256571. doi: 10.1371/journal.pone.0256571 (PMC8378680; doi:10.1371/journal.pone.0256571)

S1 Fig. Location of sample points (tower construction locations) at the La Selva Biological Station, Costa Rica. Black crosses are the sites of 55 towers in a 600 ha core area of old growth (light green). Red arrows are towers in secondary forests: 5 towers to the far west in a light tan area are in 18-year-old secondary forest, 4 towers in the yellow area in the center west are in 25-year-old secondary forest, and the 7 towers to the far east are in 36-year-old secondary forest


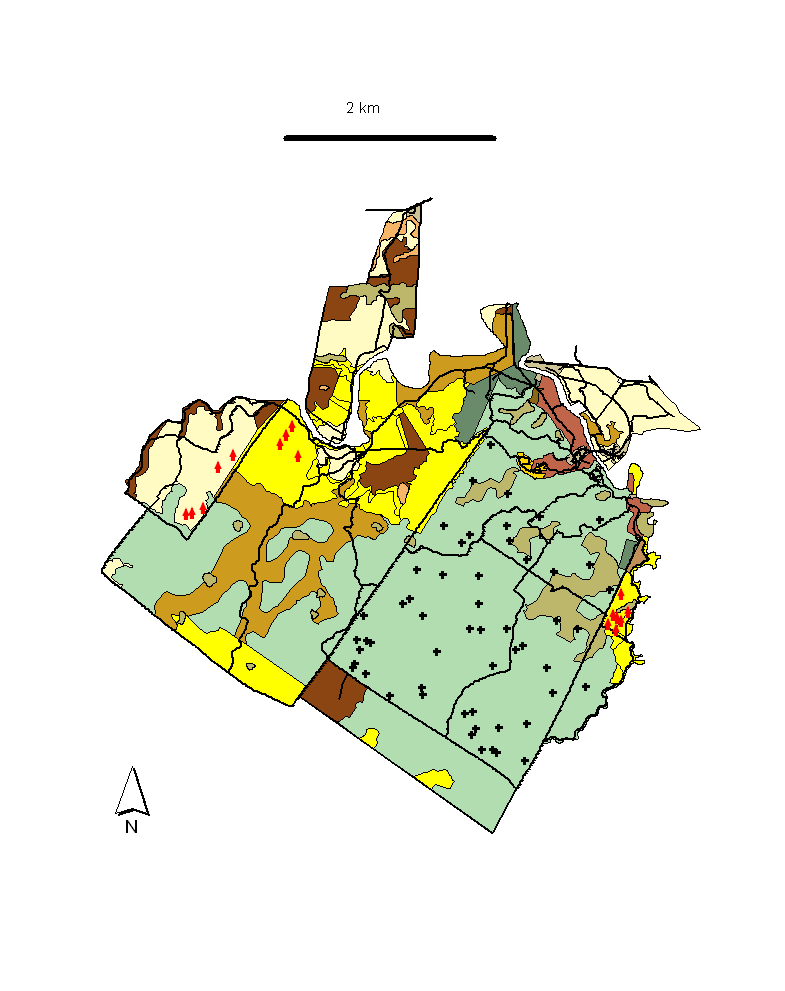

Supplement: S1 Fig — (DOCX) [file pone.0256571.s001.docx]
