# Supplementary material for: Physical structure and biological composition of canopies in tropical secondary and old-growth forests
Source: PLoS One. 2021 Aug 20;16(8):e0256571. doi: 10.1371/journal.pone.0256571 (PMC8378680; doi:10.1371/journal.pone.0256571)

S2 Fig. Canopy strata in all secondary forest towers. X = canopy strata, where leaf density is greater than an order of magnitude higher (Table 2) than in the free air spaces (indicated by ----). Towers are arranged left to right in increasing height order, and within equal tower heights, from fewer to more strata.


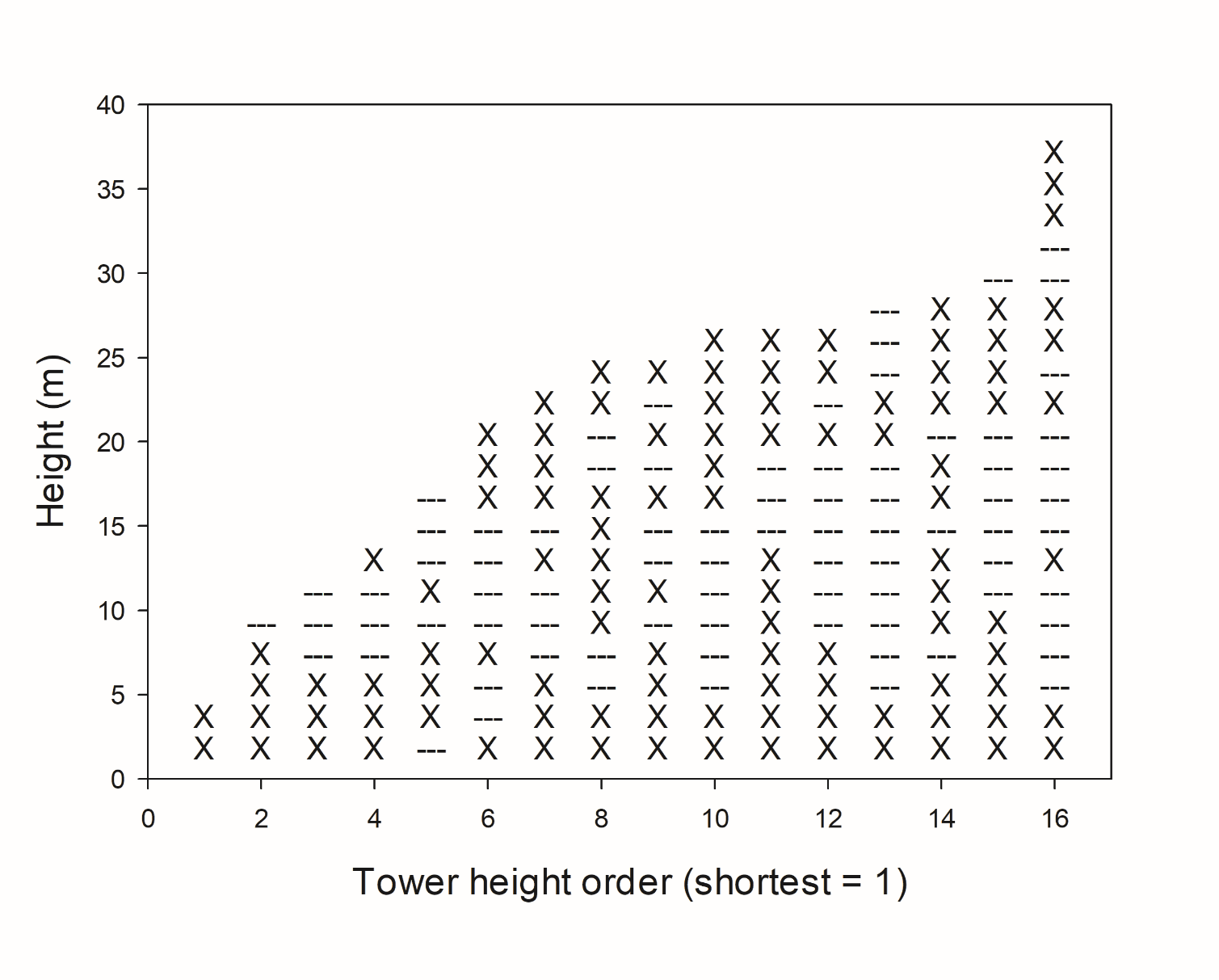

Supplement: S2 Fig — (DOCX) [file pone.0256571.s002.docx]
