# Supplementary material for: Physical structure and biological composition of canopies in tropical secondary and old-growth forests
Source: PLoS One. 2021 Aug 20;16(8):e0256571. doi: 10.1371/journal.pone.0256571 (PMC8378680; doi:10.1371/journal.pone.0256571)

S1 File. Land-used history documentation at the La Selva Biological Station, Costa Rica.

The data sources for these evaluations were aerial photographs from 1966, 1971, 1976 (2 series), 1983 (2 series), and 1988, as well as IKONOS satellite data from 2000. All images are available on request from the Organization for Tropical Studies, San Jose, Costa Rica. Place names are shown on the map at the end of this document, X_LS and Y_LS coordinates are from the La Selva internal coordinate system shown on that map. Inferred site ages are documented at the end of the text section.

Areas we evaluated for this study were:

Holdridge SF is the secondary forest area south of the Successional Plots (NE corner of La Selva) and east of the Sendero Holdridge, ~ X_LS 2000, Y_LS 1500.

El Peje SF is the secondary forest area east of the Lindero El Peje, ~ Y_LS 1800 – 2400.

Peje Pasture is the abandoned pasture west of the Lindero El Peje, ~ Y_LS = 2100-3200.

**1966 photo:**

Holdridge SF: pasture with a few remnant trees, especially at the narrow part

El Peje SF: 3 of 4 towers off image but one visible looks like it’s in at least secondary forest

Peje Pasture: off image

**1971 photos:**

Holdridge SF: mostly trees, a little brush showing through on East side

El Peje SF: all 4 towers sites clearly in forest

Peje Pasture: all 5 towers sites in pasture

**1976 photos:**

Holdridge SF: secondary forest

El Peje SF: tower sites in pasture

Peje pasture: tower sites in pasture

**1976 high-res img photo:**

Holdridge SF: all tower sites clearly in forest

El Peje SF: all 4 tower sites clearly in pasture

Peje pasture: only the 2 N-most tower sites visible, they are in clean-looking pasture

**1983 high-res img photo:**

Holdridge SF: off the image

El Peje SF: all four towers in secondary forest

Peje pasture: only north-most tower on image, it’s in clear pasture

**1983 photo.img:**

Holdridge SF: all towers in forest

El Peje SF: all 4 towers in forest

Peje pasture: all 5 towers in pasture although 3 close to some low regeneration, pasture or very low brush with <10% remnant trees and smaller trees

**1988_photo.img**

Holdridge SF: all towers in forest

El Peje SF: all 4 towers in forest

Peje pasture: all 5 towers in pasture although 3 close to some low regeneration, pasture or very low brush with <10% remnant trees and smaller trees

**2000. Iknonos_color_merge.img (image from August 2000)**

Holdridge SF: all towers in forest

El Peje SF: all 4 towers in forest

Peje pasture: regenerating pasture, lots of woody regeneration, towers look to be in areas with woody regeneration, but still what look to be fairly large areas of grass.

**Inferred Site Ages: date of pasture abandonment till time of tower construction (March-June 05)**

Holdridge SF: 2005-(between 66 and 71, say 1969) = ~36 yrs in 2005

Peje SF: 2005- (between 1976 and 83, say 1980) = ~25 years in 2005

Peje Pasture: 2005 – (1988 clean). Purchased by OTS in 1987 (McDade LA, Hartshorn GS. La Selva biological station. La Selva: Ecology and natural history of a Neotropical rain forest. 1994;379). Consider 1987 as start of regeneration, ~18 years in 2005.


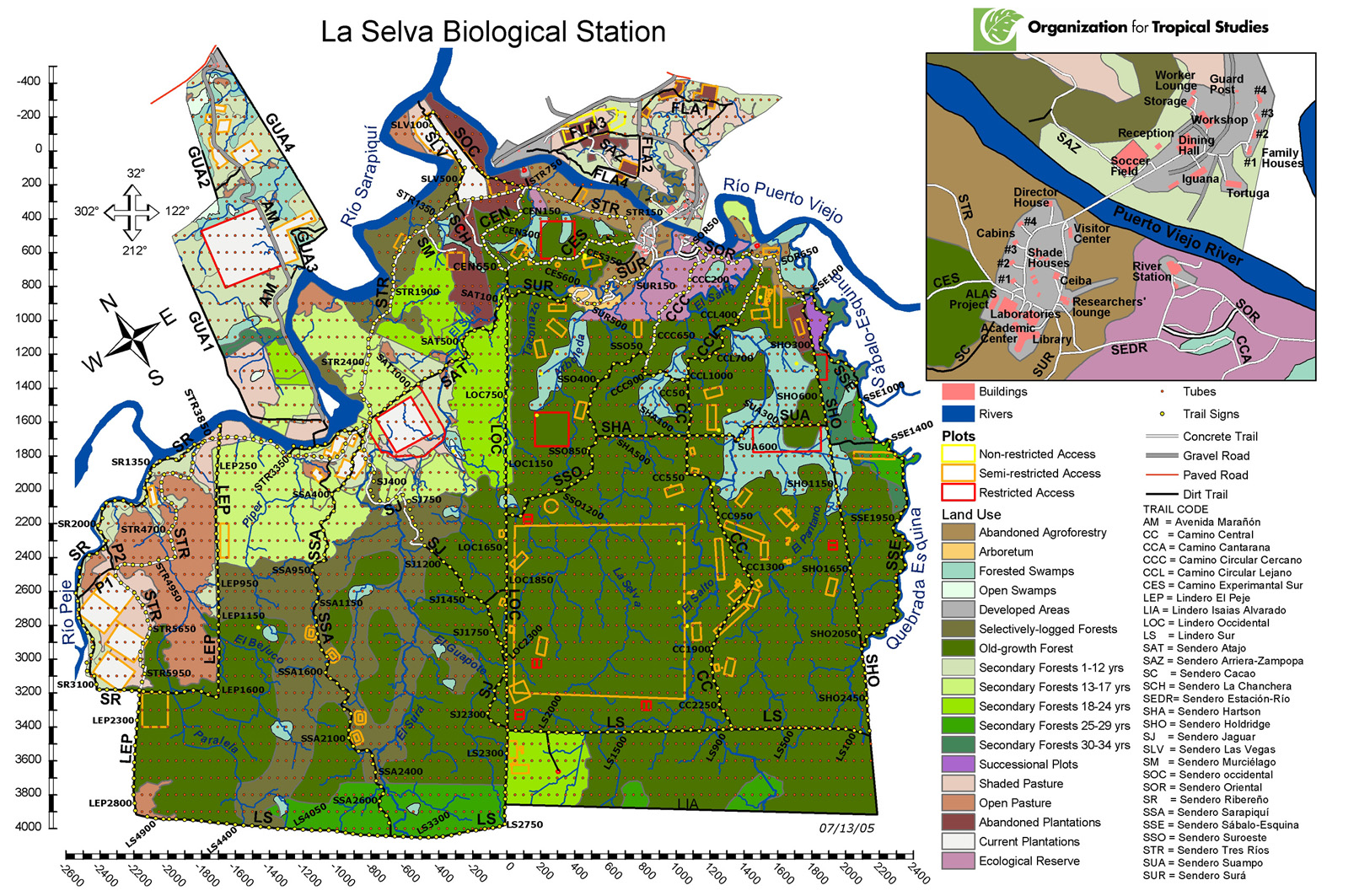

Supplement: S1 File — (DOC) [file pone.0256571.s003.doc]
